# Effect of Human and Bovine Serum Albumin on kinetic Chemiluminescence of Mn (III)-Tetrakis (4-Sulfonatophenyl) Porphyrin-Luminol-Hydrogen Peroxide System

**DOI:** 10.1100/2012/913412

**Published:** 2012-05-03

**Authors:** Sayed Yahya Kazemi, Seyed Mohammad Abedirad

**Affiliations:** Department of Basic Sciences, Sari Agricultural Sciences and Natural Resources University, P.O. Box 578, Sari 4818168984, Iran

## Abstract

The present work deals with an attempt to study the effect of human and bovine serum albumin on kinetic parameters of chemiluminescence of luminol-hydrogen peroxide system catalyzed by manganese tetrasulfonatophenyl porphyrin (MnTSPP). The investigated parameters involved pseudo-first-order rise and fall rate constant for the chemiluminescence burst, maximum level intensity, time to reach maximum intensity, total light yield, and values of the intensity at maximum CL which were evaluated by nonlinear least square program KINFIT. Because of interaction of metalloporphyrin with proteins, the CL parameters are drastically affected. The systems resulted in Stern-Volmer plots with *k*_*Q*_ values of 3.17 × 10^5^ and 3.7 × 10^5^ M^−1^ in the quencher concentration range of 1.5 × 10^−6^ to 1.5 × 10^−5^ M for human serum albumin (HSA) and bovine serum albumin (BSA), respectively.

## 1. Introduction

Serum albumins are the most extensively studied and applied proteins because of their availability, low cost, stability, and unusual ligand binding properties. Albumin is the most abundant protein in blood plasma and serves as a depot protein and transport protein for numerous endogenous and exogenous compounds. Albumin is also the main factor in contributing to the colloid osmotic pressure of the blood and has been suggested as a possible source of amino acids for various tissues. Indubitably, albumin is the most multifunctional transport protein and plays an important role in the transport and deposition of a variety of endogenous and exogenous substances in blood [1–5]. Numerous surveys were focused on determination and study of albumins by spectroscopy, isothermal calorimetery, electrochemistry, fluorescence, light scattering spectroscopy, mass spectroscopy, and chemiluminescence [6–12].

Chemiluminescence (CL) emission is generated by photochemical reactions and results from light being emitted in an exothermic chemical reaction. The attractiveness of chemiluminescence as an analytical tool lies primarily in the simplicity of detection since most samples have no unwanted background luminescence, and no optical filters are required to separate the excitation wavelengths and scatter. Since CL is directly related to the concentration of the reactants, every reaction components, including CL substrate, oxidant, catalyst, cofactor, sensitizer, enhancer, and inhibitor, can affect intensity or decay rate and be determined with low detection limit and wide linear range. More importantly, chemiluminescence is a useful analytical tool for the detection and quantification of a wide variety of biological materials such as cells, microorganisms, proteins, DNA, and RNA too [13–19]. For these reasons, significant interest has existed for application of CL techniques to the analysis of inorganic and organic substances.

**Scheme 1:**
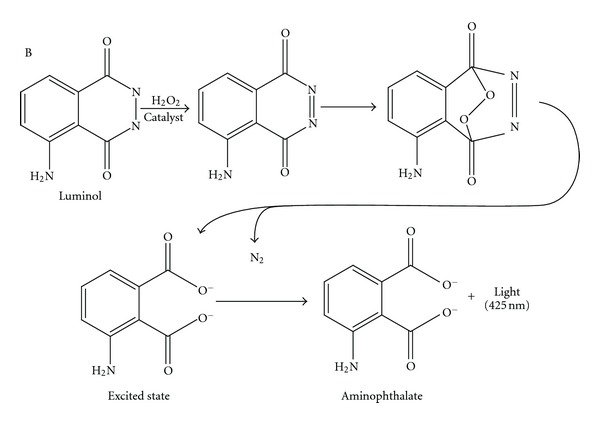
The mechanism of the CL reaction between luminol and hydrogen peroxide in the presence of catalyst.

The oxidation of luminol (3-aminophthalhydrazide) in alkaline medium is one of the most efficient CL reactions. The produced CL emission is strongly enhanced when some enzymes (e.g., horseradish peroxidase and microperoxidase) or metals (e.g., Co (II), Cu (II), Fe (II), Fe (III), and Mn (III)) are used as a catalyst. It led to produce the excited 3-aminophthalate anion, which emits light when it is relaxed to the ground state using several oxidants such as permanganate, periodate, hexacyanoferrate (III), and especially hydrogen peroxide [13, 20]. The mechanism of the oxidation and light-producing reaction of luminol is depicted in Scheme 1.

**Figure 1:**
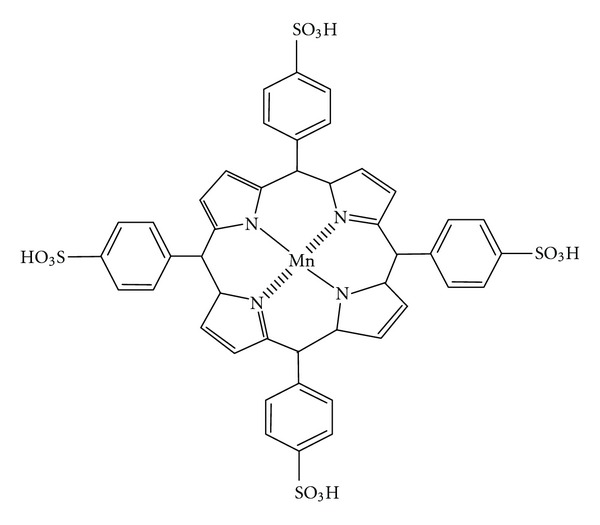
Molecular structure of MnTSPP.

In recent years, mimetic peroxidase supramolecules such as porphyrin, phthalocyanine, and their metal containing analogues have been used as catalyst in CL reaction of luminol. They have many advantages: for example, they are moderately inexpensive and have higher stability at different pH in comparison with enzymes [21–23]. In this work, manganese tetrasulfonatophenyl porphyrin (MnTSPP) as a good catalyst was employed [21] (the structure is shown in Figure 1).

A continuous interest has been focused on the use of chemiluminescence reactions for the study and detection of proteins [24–32], but as far as we know, few reports have been published to study the influence of proteins on CL kinetic. So, we are interested in investigating the effect of human and bovine serum albumin on kinetic parameters of chemiluminescence reaction of luminol-H_2_O_2_ system in the presence of manganese tetrasulfonatophenyl porphyrin as a catalyst. The investigated parameters include pseudo-first-order rise and fall rate constant for the chemiluminescence burst, maximum level intensity, time to reach maximum intensity, total light yield, and the values of the intensity at maximum CL.

## 2. Materials and Methods

### 2.1. Apparatus

Steady-state chemiluminescence measurements were carried out using spectrofluorimeter Shimadzu RF5301PC with the excitation light source being turned off. Intensity as a function of time studies was made on a luminometer equipped with photocell Hamamatsu RX-80004 which connected to a personal computer with an appropriate interface. Kinetic parameters were evaluated using KINFIT program. A Metrohm ion analyzer pH/mV meter was applied to determine pH.

### 2.2. Reagents and Solutions

Hydrogen peroxide (30% V/V), manganese tetrasulfonatophenyl porphyrin (MnTSPP), and sodium hydroxide were purchased from Fluka (Switzerland). Luminol (3-aminophtalhydrazide), human serum albumin (HSA), and bovine serum albumin (BSA) were obtained from Sigma (St. Louis, MO, USA) and used without further purification. Deionized doubly distilled water was used throughout the work.

The stock solution of luminol (0.05 M) was prepared by dissolving 88.5 mg solid in 50 mL diluted sodium hydroxide solution. H_2_O_2_ (0.1 M) was prepared in water by dissolving 0.5 mL in a volume of 50 mL. The concentration of H_2_O_2_ was determined by measuring the UV absorbance at 240 nm using *ε* 240 = 39.4 cm^−1 ^M^−1^. The MnTSPP solution was prepared by dissolving in water to the final volume of 25 mL. Stock solutions of proteins were prepared by dissolving the protein in water and stored in a refrigerator at 0–4°C. The pH of solution was adjusted by borax-NaOH buffer (0.01 M).

### 2.3. Procedure for the Study of CL System

The procedure was carried out in the following manner. The corvette was filled with 1 mL buffer, 280 *μ*L luminol, 150 *μ*L serum albumin, and 200 *μ*L porphyrin. The samples were placed in luminometer and continuously stirred up with magnetic stirrer (350 rpm). Ten seconds after luminometer began to record, the chemiluminescence reaction was initiated by injecting 250 *μ*L hydrogen peroxide to the corvette using a sampler.

## 3. Results and Discussion

### 3.1. Optimization of the Factors Affecting CL Intensity

In order to gain more concrete information to optimize CL reaction condition, an investigation was undertaken about the effect of the variables which may affect CL intensity as pH, concentration of luminol, hydrogen peroxide, and manganese porphyrin.

#### 3.1.1. Effect of Luminol Concentration on CL Intensity

**Figure 2:**
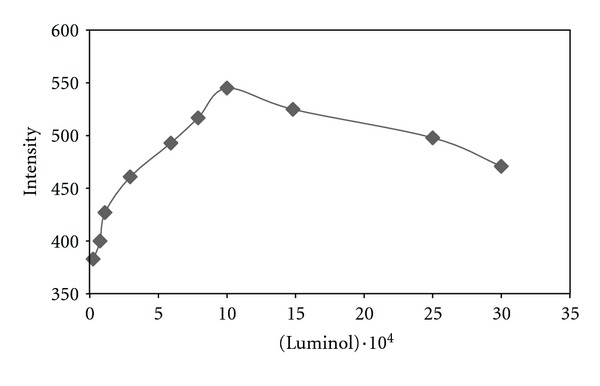
Effect of variation of the concentration of luminol on CL intensity.

Dependence of CL intensity on luminol concentration was studied at the range of 3 × 10^−5^ to 3 × 10^−3^ M (Figure 2). Since maximum intensity was observed at 1 × 10^−3^ M of luminol, it was chosen as optimal value to obtain highest CL intensity.

#### 3.1.2. Effect of Concentration of Mn (TSPP) on Intensity

**Figure 3:**
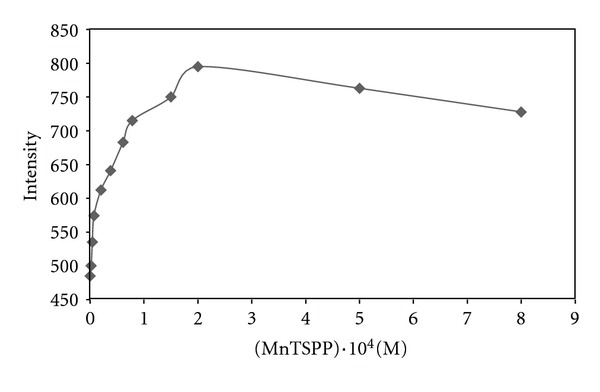
Effect of MnTSPP concentration on CL intensity.

To investigate the effect of catalyst on chemiluminescence, manganese porphyrin concentration varied from 8 × 10^−6^ to 8 × 10^−4^ M. It was found that the increase of porphyrin concentration up to 2 × 10^−4^ M leads to the increase in CL intensity, while at larger concentration, a considerable decrease in intensity was observed.  Figure 3 shows variation of intensity as a function of MnTSPP concentration.

#### 3.1.3. Effect of Oxidant on CL Intensity

**Figure 4:**
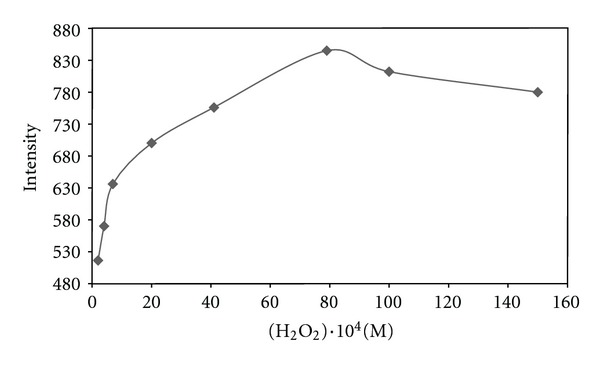
Effect of H_2_O_2_ concentration on CL intensity.

From the other point of view, to find optimal condition system, hydrogen peroxide concentration ranged from 2 × 10^−4^ to 1.5 × 10^−2^ M was investigated. It is clear from Figure 4 that a maximum CL was obtained at 8 × 10^−3^ M, while at higher or lower concentration of hydrogen peroxide, the intensity decreased.

#### 3.1.4. Influence of pH in Study of Proteins by CL

**Figure 5:**
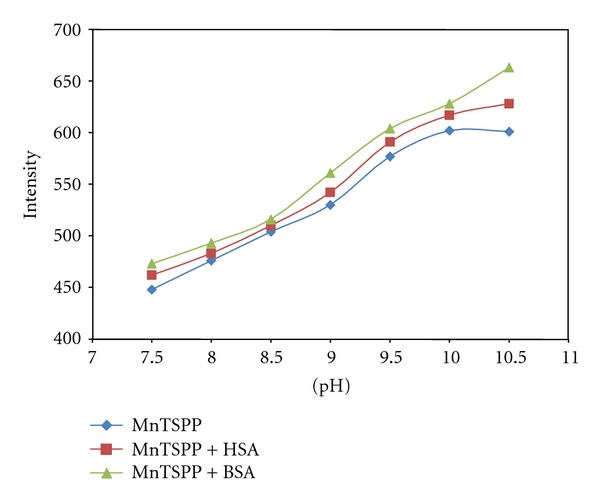
Effect of pH on CL intensity in the absence and presence of albumins.

To optimize the effect of pH of solution on CL, variation of pH which ranged from 7.5 to 12.5 was studied. It is pertinent to mention that although the maximum catalytic activity of manganese porphyrin was observed at pH 11.8–12.5, undesirable denaturing of proteins occurred at high pH, so the optimal pH in the presence of protein was evaluated at 10. The variation of CL intensity as a function of pH in the absence and presence of albumins is shown in Figure 5.

### 3.2. Study of CL Parameters Affected by Proteins

To shed light on the kinetic parameters of the chemiluminescence of luminal-H_2_O_2_-MnTSPP system in the absence and presence of serum albumins from the corresponding CL intensity versus time profiles and consecutive irreversible first-order reactions, this can be described by a simplified model which was employed [33, 34] as

(1) $$R\overset{K_{r}}{\to }X\;\overset{K_{f}}{\to }P,$$

where *R*, *X*, and *P* represent pools of reactants, intermediates, and products, respectively. Both reactions are irreversible first-order reactions. The chemiluminescence signal is proportional to the intermediate concentration. The integrated rate equation for CL intensity versus time can be obtained as follows:

(2) $$I_{\left(t\right)}=\frac{Mk_{r}}{k_{f}-k_{r}}\left(e^{-k_{r}t}-e^{-k_{f}t}\right),$$

where *I*_*t*_ is the CL intensity at time *t*, and *M* is the theoretical maximum level of intensity if the reactions were entirely converted to a CL-generating material. *k*_*r*_ and *k*_*f*_ are the pseudo-first-order rate constants for the rise and fall of the burst of CL, respectively. In addition, this pooled-intermediate model permits the estimation of intensity at the maximum CL (*J*), time of maximum intensity (*T*_max⁡_), and the total light yield (*Y*), as follows:

(3) $$\begin{array}{c} T_{\max}=\frac{\text{Ln}\left(k_{f}/k_{r}\right)}{k_{f}-k_{r}},\\ J=M{\left(\frac{k_{f}}{k_{r}}\right)}^{\left[k_{f}/\left(k_{f}-k_{r}\right)\right]},\\ Y=\overset{\infty }{\underset{0}{\int }}I_{\left(t\right)}dt=\frac{M}{k_{f}}. \end{array}$$

**Figure 6:**
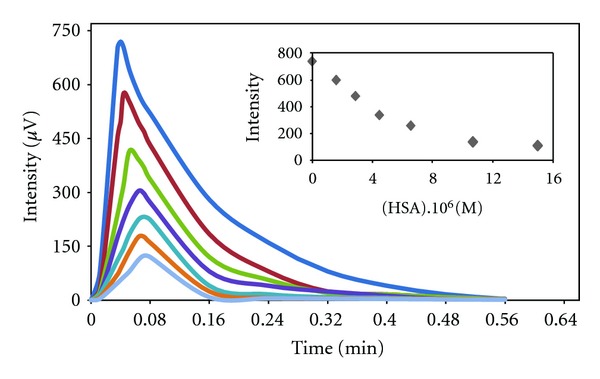
CL intensity as a function of time for reaction of luminol (1 × 10^−3^ M), MnTSPP (2 × 10^−4^ M) and H_2_O_2 _ (8 × 10^−3^ M) in the presence of varying concentration of HSA. (Inset) CL intensity as a function of HSA concentration.

**Figure 7:**
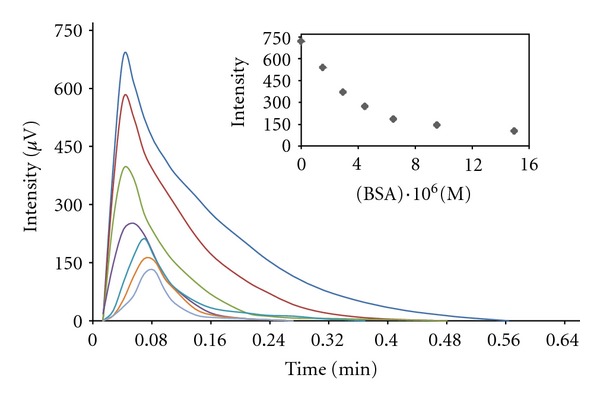
CL intensity as a function of time for reaction of luminol (1 × 10^−3^ M), MnTSPP (2 × 10^−4^ M) and H_2_O_2_ (8 × 10^−3^ M) in the presence of varying concentration of HSA. (Inset) CL intensity as a function of BSA concentration.

The *k*_*r*_,  *k*_*f*_, and other kinetic values were evaluated by computer fitting of the CL intensity-time profiles from (2), using a nonlinear least-squares curve fitting program KINFIT [35]. Then the other parameters *J*, *T*_max⁡_, and *Y* were evaluated from (3). All kinetic parameters were evaluated and presented in Table 1. Two typical response curves (i.e., light intensity versus time) for the luminol-H_2_O_2_-MnTSPP system in the absence and presence of varying concentration of HSA and BSA are shown in Figures 6 and 7, respectively. As can be seen in the absence of protein, the peak increases rapidly after mixing and reaches at maximum within 2.9 s, and the decay of light intensity from the maximum occurs during the longer periods of time (up to 38 s) via an exponential process (curve 1 in Figures 6 and 7). In the case of HSA, by first injecting (1.5 × 10^−6^ M), time to reach maximum intensity increased to 3.6 s. By increasing the concentration, *T*_max⁡_ continuously increased up to 6.18 s at 1.5 × 10^−5^ M (presented by curve 2–7, Figure 6). Furthermore, as seen in Table 1, by first addition of HSA, rise and fall rate constant decreased 1.17 and 1.07 times less than during the absence of HSA. Moreover, at higher concentration of HSA (1.5 × 10^−5^ M), rise and fall rate constant decreased 2.4 and 1.34 times less than the rise and fall in the absence of HSA. Other parameters as *M*, *J*, and *Y* decreased sharply in direct proportion of increasing HSA concentration too.

**Figure 8:**
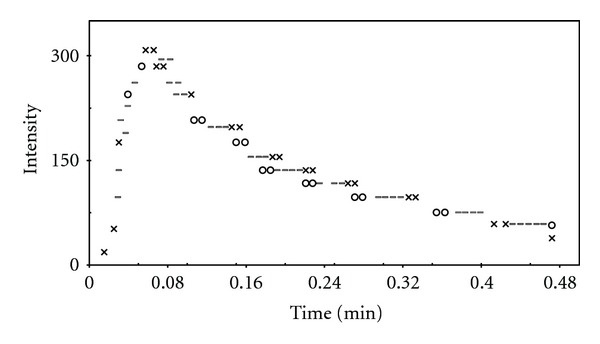
Computer fit of the CL intensity-time plot for luminol-MnTSPP-H_2_O_2_ in the presence of 4.5 × 10^−6^ M HSA: (×) experimental point, (o) calculated point, and (=) experimental and calculated points are the same within the resolution of the plot.

**Figure 9:**
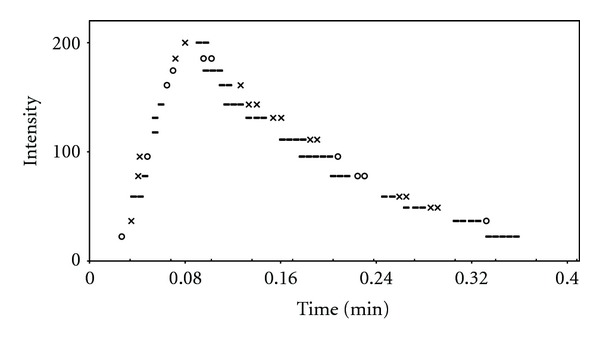
Computer fit of the CL intensity-time plot for luminol-MnTSPP-H_2_O_2_ in the presence of 6.5 × 10^−5^ M BSA: (×) experimental point, (o) calculated point, and (=) experimental and calculated points are the same within the resolution of the plot.

Figure 7 shows the effect of variation of BSA concentration on intensity versus time plots. As the results shown in Table 1, when 1.5 × 10^−6^ M of BSA is added to the cell, the decrease of rate constants was observed in comparison with the absence of BSA (1.26- and 1.04-fold for rise and fall rate constant, resp.), while *T*_max⁡_ reaches up to 3.8 s. Maximum of *T*_max⁡_ was observed at 1.5 × 10^−5 ^M, while rate constants show greatest reduction (3.57 and 1.4 times for *k*_*r*_ and *k*_*f*_, resp.). Figures 8 and 9 show typical computer fitting of the CL intensity-time plots in the presence of HSA and BSA, respectively. Fortunately, there was an adequate agreement between the calculated (*J*) and experimental (*J*_exp⁡_) values of the intensity at maximum CL. 

### 3.3. The Possible Mechanism

The mechanism of the effect of serum albumins on CL reaction can be attributed to interaction of metalloporphyrins with proteins which have been reported earlier [36–42]. Metalloporphyrin can react by pyridine and amino acids with two axial coordinations. Manganese tetrasulfonatophenyl porphyrin has several aromatic rings and four sulfonic acid groups. Because of these groups, it has high affinity to the residues of aminoacids like histidine, arginine, lysine, and the amino terminal in a protein molecule. Within CL process, metalloporphyrin reacts with hydrogen peroxide and causes to catalyze the oxidation of luminol. Interaction of metalloporphyrin and proteins can inhibit peroxidase activity of metalloporphyrin to produce 3-aminophtale and hence emitting light (Scheme 1). Table 1 shows that the rate constants and *J* dramatically decreased as the protein concentration increased.

### 3.4. Study of Quenching CL by Serum Albumins

As it is obvious in Table 1, a considerable decrease in intensity of chemiluminescence system occurred as the concentrations of HSA and BSA are increased. So we decided to study the term of quenching chemiluminescences which have been described by Fletcher and Heller [43]. In the presence of quencher, chemiluminescence intensity reduced from *I*_*o*_ to *I*_*Q*_. The ratio of Cl intensity was reduced directly with quencher concentration (*Q*) increasing, as stated by Stern-Volmer plots equation [44]

(4) $$\frac{I_{o}}{I_{Q}}=1+k_{Q}\left[Q\right],$$

where *k*_*Q*_  is the Stern-Volmer quenching constant.

**Figure 10:**
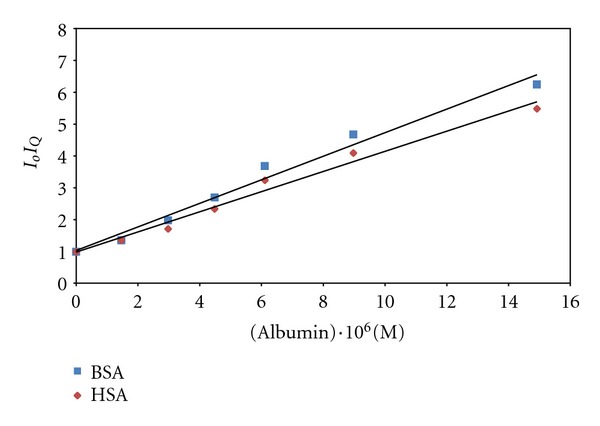
Stern-Volmer plots of *I*_*o*_/*I*_*Q*_ versus concentration of albumins.

Based on (4), a plot of *I*_*o*_/*I*_*Q*_  versus [*Q*] will result in a linear graph with an intercept of 1 and a slope of *k*_*Q*_. For a measurement system based on quenching, *k*_*Q*_ should be as large as possible. Plots of *I*_*o*_/*I*_*Q*_ versus concentration of HSA and BSA as quenchers were evaluated and presented in Figure 10. The resulting regression equations in the concentration range from 1.5 × 10^−6^ to 1.5 × 10^−4 ^M are as follows:

(5) $$\begin{array}{c} \frac{I_{o}}{I_{Q}}\;=\;0.953+\;0.317\times 10^{6}\;\left[\text{HSA}\right]\;r^{2}=0.9792,\\ \frac{I_{o}}{I_{Q}}=\;0.991+\;0.370\times 10^{6}\;\left[\text{BSA}\right]\;r^{2}\;=0.9821. \end{array}$$

Decrease in *k*_*r*_, *M*, *J*, and *Y* and also increasing time to reach maximum intensity vividly verified strong inhibiting of chemiluminescence of luminol-metalloporphyrin system by albumins. It should be noted that the protein detection by the quenched chemiluminescence of luminal-H_2_O_2_-Mn TSPP system is based on a reduction in the CL signal due to interaction of porphyrin with proteins. As it is expected, the noise of this signal should be reduced as much as possible to achieve a favorable signal-to-noise ratio for the quenched chemiluminescence. This can be obtained easily at a high *I*_*o*_ value, since a greater dynamic range is then obtainable. It can be concluded that metalloporphyrin chemiluminescence of luminol is appropriate for the quenched chemiluminescence mode in the case of proteins.

## 4. Conclusion

In this work, the effects of human and bovine serum albumin on kinetic parameters of chemiluminescence of luminol catalyzed by manganese tetrasulfonatophenyl porphyrin were studied. The investigated parameters involved pseudo-first-order rise and fall rate constant for the chemiluminescence burst, maximum level intensity, time to reach maximum intensity, total light yield, and values of the intensity at maximum CL. It has been observed that as the albumins are interacting with metalloporphyrin, the intensity of chemiluminescence was decreased. Moreover, the Stern-Volmer plots for quenched chemiluminescence system in the presence of albumins were also evaluated.

## Figures and Tables

**Table 1:** CL kinetic parameters evaluated from computer fitting of the CL intensity-time for luminol-MnTSPP-H_2_O_2_ in the presence of serum albumins.

Parameters	Concentration (M)	*k*_*r*_ (min^−1^)	*k*_*f*_(min^−1^)	*M*	*J*	*J*_exp⁡_	*T*_max⁡_ (min)	*T*_exp⁡_ (min)	*Y*
	0	80 ± 4	2.6 ± 0.44	932 ± 2	646	728	0.044	0.049	358
	1.5 × 10^−6^	68 ± 2	2.42 ± 0.08	843 ± 3	547	573	0.051	0.060	348
	3 × 10^−6^	64 ± 3	2.36 ± 0.23	630 ± 2	402	433	0.053	0.066	183
HSA	4.5 × 10^−6^	58 ± 5	2.31 ± 0.03	438 ± 6	277	313	0.058	0.070	189
	6.5 × 10^−5^	47 ± 1	2.18 ± 0.68	335 ± 3	206	225	0.068	0.082	153
	9.5 × 10^−6^	39 ± 4	2.1 ± 0.81	276 ± 3	168	175	0.079	0.086	131
	1.5 × 10^−5^	33 ± 2	1.94 ± 0.2	204 ± 2	177	133	0.091	0.103	105

	1.5 × 10^−6^	63 ± 7	2.49 ± 1	776 ± 4	523	555	0.053	0.064	311
	3 × 10^−6^	53 ± 5	2.42 ± 0.35	551 ± 2	369	375	0.061	0.073	227
BSA	4.5 × 10^−6^	41 ± 5	2.32 ± 0.71	366 ± 2	244	271	0.074	0.08	157
	6.5 × 10^−5^	36 ± 4	2 ± 0.11	297 ± 1	174	199	0.085	0.097	148
	9.5 × 10^−6^	28.6 ± 6	1.93 ± 1.2	208 ± 2	122	157	0.101	0.110	107
	1.5 × 10^−5^	22.4 ± 6	1.85 ± 0.25	157 ± 3	93	115	0.121	0.132	84
